# Medical Exercise Therapy is Effective After Arthroscopic Surgery of Degenerative Meniscus of the Knee: A Randomized Controlled Trial

**DOI:** 10.4021/jocmr1100w

**Published:** 2012-11-11

**Authors:** Havard Osteras, Berit Osteras, Tom Arild Torstensen

**Affiliations:** a Department of Physical Therapy, Sor-Trondelag University College, Faculty of Health Education and Social Work, Trondheim, Norway; b Sor-Trondelag University College, Trondheim, Norway; c Holten Institute, Lidingo, Sweden

**Keywords:** Rehabilitation, Physical therapy, Meniscus

## Abstract

**Background:**

There is no consensus in postoperative rehabilitation regimen for people who had undergone surgery for degenerative medial meniscus damage. The aim of this study was to examine whether it is beneficial to undergo postoperative physiotherapy after surgery for these patients.

**Methods:**

A prospective randomized controlled clinical trial. Over a 4 month period, 70 participants were randomly assigned into a high repetitive, high dosage medical exercise therapy group (EG) (n = 36) or into a control group (CG) (n = 34). Pain was a composite score of visual analogue scale (VAS). Function was measured with a functional assessment questionnaire (KOOS). Muscle strength was measured with a five repetition maximum test of quadriceps femoris.

**Results:**

Prognostic variables were similar between the groups at baseline. Five (7%) people dropped out during the treatment period. The EG achieved significantly better outcome effects than the CG at pain (VAS reduced 1.9 in EG and 0.6 in CG) and function (KOOS decreased 18 in EG and only 6 in CG).

**Conclusions:**

For people who have undergone surgery for degenerative meniscus damage, postoperative high repetitive, high dosage medical exercise therapy is an efficient treatment alternative compared to no rehabilitation.

## Introduction

Middle-aged people with degenerative meniscus injuries account for a large group of patients. They have knee pain, swelling and impaired function [1]. Many knee injuries occur without any trauma in both physically active people and older people, and can be part of early osteoarthritis [1]. Partial arthroscopic surgery of the meniscus is the common surgical procedure in patients with meniscus injury. Postoperatively, many patients report less pain, better function and better quality of life [2]. Despite reduction in knee pain and improved knee function, Roos et al [3] showed that 3 months after knee surgery, a majority of patients had reduced physical activity and 38% were not physically active, compared with only 9% before the operation.

During surgical treatment for a torn meniscus, the damaged part of the meniscus is removed from the knee, leaving behind as much healthy tissue as possible. Despite this procedure is minimally invasive in nature, studies have shown that patients who have partial meniscectomy surgery experience pain and swelling, leading to reduced function and knee-related quality of life [3, 4]. There is no consensus in the literature whether these patients need formal physical therapy after this kind of surgery. In addition, many of the existing studies concerning postoperative physical therapy have lack of detailed descriptions of the interventions. Goodwin and Morrissey [5] concluded in a review that those patients who have undergone an uncomplicated arthroscopic partial meniscectomy, physical therapy is not necessary, as it will have little or no effect on their returns to activities of daily living. However, they also points to the general suffering from methodological problems in the studies included, and again, the interventions completed were poor described and lasted in general only 4 - 6 week postoperatively.

At present, there is no universal agreement as to what rehabilitation protocol is best. Physical activity is well documented as effective treatment for patients with knee degeneration to improve function and reduce pain, both in subacute and long-term patients [6]. However, there is a lack of evidence regarding effect of active postoperative rehabilitation after meniscus repair [7]. There exists no consensus from studies or best practice among orthopedic surgeons whether postoperative rehabilitation is necessary or not.

Several studies point out that patients with knee pain develop compensatory movement strategies in functional tasks, probably because of pain, fear of pain or muscle failure [8]. It appears that these compensatory strategies are permanent, even when the relevant function tasks no longer trigger pain, and therefore involves dysfunction. In order not to provoke any inflammation, the exercises should be as painless as possible [9]. One way to perform this can be using numerous pain-free, repetitive movements that are deemed beneficial to the healing of local tissue damage [10].

Medical exercise therapy (MET) is a well-known and recognized treatment approach. MET is defined as a treatment approach with its own criteria; “The therapist is in the exercise room supervising the subjects while he/she are treated with graded exercise therapy. The therapy consists of one hour active therapy. The history taking and clinical assessment is the basis for designing an individual exercise program, and the subjects are in a group setting consisting of maximum 5 subjects” [11]. Specially designed exercise equipment is used for grading exercises [11]. MET is a system for applying progressive resistance exercises were the aim is to use exercise treatment as a “pain treatment” to decrease the subjects’ pain experience and to improve impaired function. The treatment objective is to perform approximately 10 exercises resulting in more than 1,000 repetitions during each treatment. The high number of repetitions in sets also stimulates improved coordination and increase range of motion. The grading of the exercises makes it possible and imperative to perform these exercises close to a pain free threshold, within a comfortable range of motion, along with emphasis on good coordination.

The aim of this study was to compare clinical effects of two approaches after arthroscopic surgery in patient with degenerative meniscus; supervised high repetitive, high dosage medical exercise therapy versus no rehabilitation program.

## Materials and Methods

**Figure 1:**
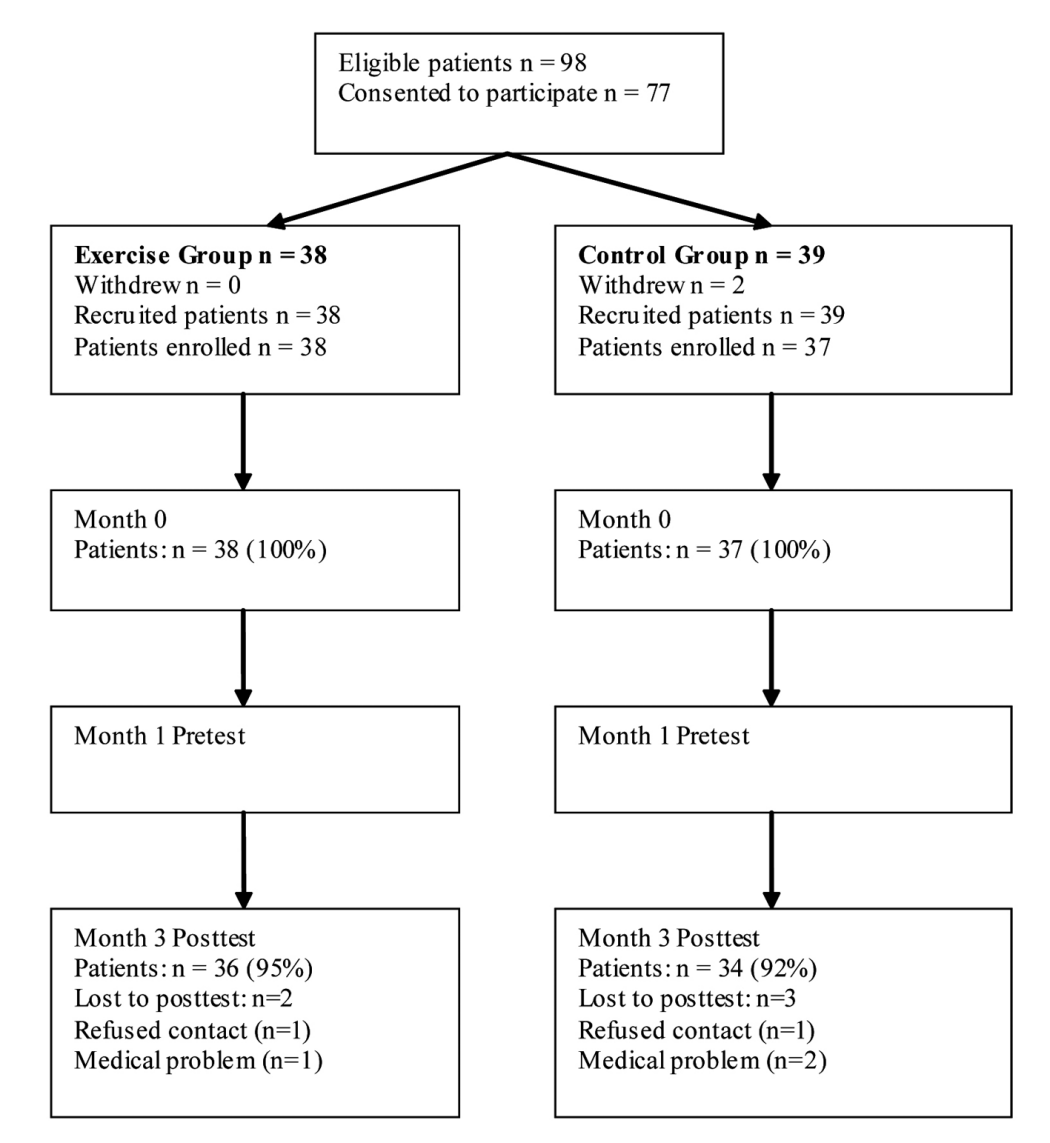
Subject flow diagram of the patients.

The present study was a randomized, controlled clinical trial with two groups; a high repetitive, high dosage medical exercise therapy (EG), and a control group that were instructed not to participate in a rehabilitation program (CG) (Fig. 1). A computer-generated randomization schedule was used, with annotations for treatment according to medical exercise therapy or no postoperatively rehabilitation. The same investigator, not involved in the randomization procedure, prepared all envelopes in the study. To maintain the blinding of the study, four different well experienced physical therapists with accreditation in MET did the testing and the exercise intervention respectively. Ethical approval was acquired from the Human Review Committee (Trondheim, Norway) and all participants gave their written consent to participate in the study after receiving written information about the study.

Inclusion criteria were subjects with knee pain for more than 2 - 3 months, no considerable acute first appearance, 35 - 60 years, functional limitations in everyday life or physical activity making the assessor evaluate the patient eligible for arthroscopic partial meniscectomy, MRI showing degenerative meniscus tear. Exclusion criteria were ACL rupture, those requiring acute trauma surgeries, knee ligament injuries, osteoarthritis grade 3-4 [12], haemarthroses and acute cases of locking knee, and symptomatic pain in contrary extremity. Further exclusion criteria were other musculoskeletal comorbidities severely affecting lower extremity muscle function overriding the symptoms from the knee, comorbidities excluding physical activities and exercise, not able to speak or read the language of interest, drug abuse or mental problems.

Patients were recruited by orthopedic surgeons in three hospitals in the middle of Norway over one year. The intervention exercise was considered not to cause deterioration of the injury or pain and was implemented in line with the known training principles used by physical therapists. All participation was based on informed consent, voluntariness and the right to withdraw from the study without further consequences.

### Outcome measures

The primary outcome is pain; a subjective score measured with a visual analog scale (VAS) at rest recorded on a 0 - 100 mm line. The extreme limits were marked by perpendicular lines using the verbal descriptors of “no pain” and “worst pain I can imagine”. The VAS has been shown to be a reliable tool for measuring pain [13]. The secondary outcome was a self-reported composite measure; “Knee Injury and Osteoarthritis Outcome Score” (KOOS) comprising five different subscales; a) pain, b) other symptoms, c) activities in daily living, d) function in sport and recreation and e) knee related quality of life (QOL) [14]. All items have five possible answer options scored from zero (no problems) to four (heavy problems). The scores were transformed to a 0 - 100 scale, where 100 represent no knee-related problems. The KOOS is a valid and reliable patient-relevant questionnaire for patients with knee injury and knee osteoarthritis [14]. To detect an average difference between individuals and between groups, a minimal perceptible improvement was set to ten points. KOOS was registered at baseline and post test during this trial.

From zero to one month post-operatively the EG patients received treatment involving information about pain relief, range of motion exercises, quadriceps training exercises with very low load, a few minutes bicycling with low load, gait exercises to avoid limping. One month postoperatively and after the intervention period (3 months) all patients answered the questionnaires and completed the muscle test at the same day. Prior to the test, subjects warmed up on a stationary bicycle for approximately 10 minutes. A leg extension bench for evaluation of quadriceps muscle strength deficits was included, with 5 repetition maximum (RM). The reliability for the muscle test has previously been reported to be satisfactory [15].

### Intervention

Standard arthroscopic partial meniscectomy (NGD 11) was applied as surgical intervention. It was carried out at St Olav University Hospital and Teres Rosenborg Clinic in Trondheim, Norway, and performed on patients that fulfilled the inclusion criteria. Normal procedures for this surgery were followed at the respective hospitals.

The exercise program was further developed for this particular study, with focus on coordination, circulation and muscle function. Improved muscle strength and coordination could potentially have positive influence on knee symptoms, function and finally the progression of osteoarthritis. The program was conducted in the most possible degree, allowing for individual differences due to performance and progression. Based on clinical experience, the intervention period was three months, and the subjects performed the exercise program 3 times per week at the physiotherapy institutes. Symptoms and clinical findings were the basis for choosing individual starting positions, range of motion, and weight resistance for each exercise. The treatment goal in the exercise group was to perform three sets of 30 repetitions. The program was a combination of the global aerobic exercises using a stationary ergometer bicycle, and the semiglobal and local exercises to modulate pain and increase range of motion using specially designed exercise equipment. This included wall pulleys, and quadriceps and hamstrings muscle strength training apparatus (Table 1).

**Table 1:** The Exercise Program for the Medical Exercise Therapy Group Performed During the 12 Weeks

	Exercise	Dosage
1	Stationary bicycling	10 - 20 minutes
2	Deloaded^*^ step up	3 × 30 repetitions
3	Deloaded^*^ knee extension	3 × 30 repetitions
4	Squat	3 × 30 repetitions
5	Stationary bicycling	10 minutes
6	Deloaded^*^ step down	3 × 30 repetitions
7	Loaded knee extension, open chain	3 × 30 repetitions
8	Deloaded^*^ knee extension	3 × 30 repetitions
9	Stationary bicycling	10 minutes

^*^Exercises in closed kinetic chain with less than body weight.

Each treatment in the EG, started with 10 - 20 minutes aerobic work on a stationary ergometer bicycle. Half way through the exercise program, after four exercises each of three sets of 30 repetitions, the subjects cycled for 10 minutes. Again after the last four exercises, the subjects did another 10 minutes on the stationary ergometer cycle. The intensity during cycle exercises was moderate to high, i.e. a heart rate frequency of 70-80% of the maximal heart rate, measured by heart rate monitors. The hypothesis was that the global exercises are important to stimulate the body’s own pain modulating system through the gate control mechanism in the posterior horn of the spinal cord and for the release of the endogenous neuropeptides in the central nervous system.

### Statistics

For sample size calculation, the significance level was set at 5.0%, with a power of 90% and an SD of 3.2 points on VAS, as presented by [16]. Total number of subjects to randomize into two groups was estimated to be 70. The calculated drop-out ratio was 10%. The statistical analysis was performed using the commercial software package PASW for Windows (release 19.0). Descriptive statistics were performed for demographic variables. Normal distributions of outcome variables were estimated by the Komokolov-Smirnov test. Within and between mean group differences were analyzed by using a general linear model. Intervention (group allocation) and time (between pre- and posttest) were main effects and baseline values of the primary outcomes were applied as covariates. Bonferroni was used to estimate main group differences. An intention-to-treat analysis was not used because of very low drop-out rate. Each participant’s compliance was determined by averaging the compliance reported on compliance logs.

## Results

All possible efforts were made to enhance compliance and adherence with the program. The subjects completed 82% of the rehabilitation program. Baseline characteristics of the study population are given in Table 2. All outcomes were normally distributed at pretest.

**Table 2:** Baseline Characteristics of the Study Population. Mean (SD) Values, Unless Otherwise Noted

	Exp group (n = 36)	Con group (n = 34)	Total (n = 70)
Age, years	46.3 (8.3)	46.3 (8.9)	46.3 (8.6)
Weight, kg	79.6 (9.8)	80.1 (9.8)	79.9 (9.7)
Height, cm	177.2 (7.6)	176.3 (6.2)	176.7 (6.9)
Duration of symptoms, years	2.1 (2.3)	2.1 (1.6)	2.1 (2.0)
Gender (% female)	12 (33)	11 (32)	23 (33)
Osteoarthritis 1^*^ (%)	9 (25)	6 (18)	15 (21)
Osteoarthritis 2^*^ (%)	3 (8)	7 (21)	10 (14)

^*^ According to the Outerbridge classification (Cameron et al 2003).

Degenerative and/or other chondral lesions were found in 25 cases; 12 in the EG group and 13 in the CG. According to the Outerbridge classification [17] there were 15 patients with grade I (softening of the surface), and 10 patients with grade II (partial-thickness defects less than 1.5 cm). Grade III and IV patients were excluded from the study, 12 (48%) of the degenerative chondral lesions with unstable flaps were treated by debridement with a motorized shaver, 23 (92%) of these debridements concerned the medial aspect of the joint.

Twelve weeks after surgery, both groups showed significantly (P < 0.05) better scores on VAS and KOOS than after 1 month postoperatively. Both after 1 and 3 months postoperatively, the EG showed significantly (P < 0.05) less pain (VAS) and increased function (KOOS), compared to the CG (Table 3).

**Table 3:** Mean (SD) Outcome Values in the Groups at Baseline and After 3 Months, Mean (SD) Within Groups Changes, and Adjusted Mean (95% CI) Difference Between Groups After 3 Months

	Outcome within groups^†^ Pre-test		Groups between groups^‡^ Post-test		Difference		Adjusted difference
	Exp (n = 36)	Con (n = 34)	Exp (n = 36)	Con (n = 34)	Exp		Con
VAS	3.3 (2.1)	2.9 (1.5)	1.4 (1.4)	2.3 (1.3)	-1.9 (1.6)	-0.6 (0.6)	-1.1^*^ (-1.5 to -0.6)
FiveRM	11.6 (4.3)	12.5 (5.2)	20.2 (5.4)	14.5 (5.2)	8.6 (3.8)	2.0 (2.3)	6.5^*^ (5.0 to 8.0)
KOOS	48.0 (21.7)	43.4 (22.9)	30.0 (17.7)	36.9 (23.1)	-18.0 (10.9)	-6.5 (6.4)	-10.7^*^ (-14.7 to -6.7)

Abbreviations: Exp, experimental group (EG); Con, control group (CG). ^*^ The adjusted difference between groups were all significant at P < 0.01, all in favor of the experimental group. ^†^ Post minus pre; change scores. ^‡^ Post-test scores of the primary outcomes were all adjusted for baseline values.

## Discussion

The aim of this study was to compare clinical effects of two approaches after arthroscopic surgery in patient with degenerative meniscus. The results showed that supervised high repetitive, high dosage medical exercise therapy reduces pain and increases overall function significantly compared to no supervised rehabilitation. Since there is no consensus from orthopedic surgeons or researchers in this field whether postoperative rehabilitation is necessary or not, the present results show that a structured exercise program might be a contribution in that debate. There are a few reported trials on postoperative physiotherapy, but that is primary in younger people with acute meniscus damages. In the present study, we hypothesize the importance of postoperative physiotherapy, as the degenerative meniscus patients might be in the “osteoarthritis category”, in which the importance of exercise rehabilitation is well documented [18].

Arthroscopy can be an effective short-term as well as long term-term treatment, especially in those without articular cartilage damage [4]. Patients with non-degenerative meniscal tears are more satisfied with their knee function after arthroscopy than those patients with degenerative meniscal tears [19]. There is an increased risk of developing tibiofemoral osteoarthritis, verified by radiography, for individuals older than 40 years old after meniscectomy, and therefore exercise treatment over several months could be recommended as the first treatment choice.

Ericsson et al [20] found that quadriceps strength is reduced in the meniscectomized leg compared with the nonoperated leg 4 years after surgery. They suggest that the relative quadriceps weakness significantly affects objective and self-reported knee function, pain, and quality of life, indicating the importance of restoring muscle function after meniscectomy in middle-aged patients. Ten to twenty years after surgery for cruciate ligament and/or menisci damage every second patient develops osteoarthritis [21], though it is not known why such damages may be a risk factor for osteoarthritis. The present study did not take into account what kind of physical activity level or professional work load the patients aimed to return to. Further studies should shed more light on this.

When using evaluation tools, it is essential to consider reasonable guidelines for determining a “clinically significance change”. Statistical significance is a necessary condition for proving treatment effectiveness, however, in large sample studies small differences may be statistically significant, but the results are not clinically relevant [22]. The effect size is defined by Cohen (p 9) [22] as “the degree to which the phenomenon is present to the population”, indicating that effect size represents a dimensionless number, void raw units. Based on Cohen’s guidelines, it appears that effect sizes of 0.2 or less would not be useful indicators of real clinical change for VAS. In the present study, VAS changes were statistically significant compared between pre- and posttest; however, the clinical significance may not be present. For five RM there exists no consensus regarding clinically significance. In KOOS, Roos and Lohmander [23] suggests a clinically significance level of 10 points or more of improvement or decline. In the present study, the EG had a decrease in 18 units, while the CG only decreased 6.5; both a statistically and a clinical difference.

With the realization of the meniscus as a vital structure to proper knee integrity, function, and longevity, the sports medicine and orthopedic communities have shifted the focus of conservative and surgical treatment to that of meniscal conservation. Menisci play a vital role in load transmission, shock absorption and joint stability. Osteoarthritis is a whole joint disease, where meniscal cells may play an active role in the development of osteoarthritis [3]. Future research with long-term follow-up design may describe whether a guided rehabilitation period may delay the development of osteoarthritis. The intervention in the EG emphasized a relatively high-repetitive and high volume of endurance training, trying to increase local circulation in the injured joint, as well as increase joint functional stability during many repetitions in pain-free range of motion. However, there is to our knowledge no evidence whether higher external resistance could be beneficial in meniscal patients. Clinical experience suggests that therapists should be careful with using high external load, leading to few repetitions per set, in this group of patients.

### Limitations

There was no collection of information as to what the CG group did of potential biasing activity during the experimental period. Many patients know that physical activity and strength training might be beneficial after surgery for meniscus damage. However, since the number of repetitions were so high over three months postoperatively, we do not think this would affect the result significantly. Another limitation is the lack of blinded assessors. However, this was a multicenter study with four physiotherapists, which reduces the possible biases of internal validity in such a clinical trial. Two outcome measures were self reports (pain, KOOS) so there could not be blinding to group allocation, though we acknowledge the lack of blinding as a limitation. It should also be noted that participants in the exercise group may have been involved in other types of therapy or treatment in addition to exercises. This was not tracked and should be recognized as another study limitation.

There is a strong need for further research in the field dealing with the necessity of postoperative rehabilitation. There is also a lack of evidence regarding what kind of postoperative rehabilitation physical therapists should emphasize. An effective rehabilitation might reduce sick leave and thus public costs, as well as improve the quality of live and return to everyday living. Emphasis should be put on clinical trials with a follow-up design, comparing difference rehabilitation protocols. Measurements of e.g. the release of endogenous neuropeptides could be done. Further trials should be adequately powered and address blinding of outcome assessor. Future research may also describe the cost-benefit-analysis in this group of patients, due to sick leave from work and the related need for health care. Despite of the mentioned methodological limitations, we claim that the present study has a certain amount of generalizability.

### Conclusion

The results from the present study showed that supervised high repetitive, high dosage medical exercise therapy reduces pain and increases knee function better and faster than postoperative patients not participating in a professionally guided rehabilitation program. The present study adds some knowledge to this field, and it seems like there might be a significant rehabilitation potential for people who have had a surgery for meniscus damage by the implementation with the principles of supervised high repetitive, high dosage medical exercise therapy.
